# Distal Oblique Bundle and Membranous Thickening: Morphology and Integration with the Triangular Fibrocartilage Complex

**DOI:** 10.3390/diagnostics15212728

**Published:** 2025-10-28

**Authors:** Yuri Seu, Seong-Kyu Choi, Jin Seo Park, Hongtae Kim, Mi-Sun Hur

**Affiliations:** 1 Daegu Catholic University School of Medicine, Daegu 42472, Republic of Korea; 2 Department of Anatomy, Daegu Catholic University School of Medicine, Daegu 42472, Republic of Korea; 3 Department of Anatomy, Ajou University School of Medicine, Suwon 16499, Republic of Korea

**Keywords:** distal oblique bundle, membranous thickening, interosseous membrane, distal radioulnar joint, triangular fibrocartilage complex

## Abstract

**Background:** The distal oblique bundle (DOB) of the interosseous membrane (IOM) has been recognized as an important stabilizer of the distal radioulnar joint (DRUJ). However, its prevalence, morphology, and distal attachments—particularly its relationship to the articular disc and the extensor carpi ulnaris (ECU) tendon sheath—remain inconsistently described. Clarifying these anatomical details is essential for understanding DRUJ stability and guiding surgical reconstruction. **Methods:** The distal IOM was examined in 48 specimens from 24 embalmed Korean cadavers. In 46 dissected specimens, the presence, morphology, and attachment sites of distal interosseous structures were documented, and attachment levels were measured. In 38 specimens, attachment to the articular disc was assessed. In addition, serial transverse sections from one cadaver were analyzed to confirm three-dimensional relationships. **Results:** Two morphological patterns were identified: a distinct DOB (21/46, 45.7%) and, when absent, a membranous thickening of the distal IOM (25/46, 54.3%). The mean attachment level was 39.1 ± 9.7 mm for the DOB and 25.4 ± 4.8 mm for the membranous thickening. Both structures assumed an oblique orientation, fanning palmarly toward the capsule and articular disc and dorsally toward the ECU tendon sheath and dorsal septum. In 26 of 38 specimens (68.4%), these structures attached to the proximal palmar portion of the articular disc. Serial transverse sections confirmed this oblique configuration, linking palmar and dorsal stabilizers of the DRUJ. **Conclusions:** The distal IOM consistently forms specialized structures—either a DOB or a membranous thickening—that integrate with the triangular fibrocartilage complex. By bridging palmar and dorsal stabilizers, these structures contribute to joint congruency and load transfer during forearm rotation. A refined anatomical understanding of these patterns provides clinically relevant insights for surgical preservation or reconstruction, with the potential to improve outcomes in patients with chronic DRUJ instability.

## 1. Introduction

The interosseous membrane (IOM) of the forearm is a syndesmotic structure that connects the radius and ulna [1]. It serves as a biomechanical stabilizer, transmitting load between the two bones while contributing to both longitudinal and rotational stability of the forearm [2,3]. Anatomically, the IOM is primarily divided into distinct components, including the proximal oblique cord, the distal oblique bundle (DOB), and the central band [1,4,5,6]. Each component has characteristic fibre orientations and attachment sites, reflecting specialized roles in forearm mechanics.

In particular, the DOB, which constitutes the distal part of the IOM of the forearm, has been the focus of several anatomical and biomechanical studies for its relationship to the distal radioulnar joint (DRUJ) and its role in joint stability [5,7,8,9,10,11,12,13,14,15,16,17,18,19,20]. The reported prevalence of the DOB, investigated through cadaveric dissection, ultrasonography, and MRI studies, has varied widely, ranging from 29% to 87.5% of specimens [5,8,9,12,14,15,16,17,18,19,20,21]. Morphologically, the DOB has exhibited diverse configurations, such as a linear bundle either separate from or within the distal membranous portion of the IOM, as well as fan-shape and wide types [9]. It has been reported that the DOB originated from the distal one six of the ulnar shaft and coursed obliquely toward the radius, inserted into the inferior rim of the sigmoid notch of the radius, capsular tissue of the DRUJ, dorsal and palmar radioulnar ligaments of the triangular fibrocartilage complex (TFCC) [5,9,10,11,15].

Biomechanical studies have highlighted the functional importance of the DOB in stabilizing the DRUJ during pronation and supination. Watanabe et al. (2005) [7] demonstrated that the DOB acts as a dorsal–volar stabilizer of the DRUJ, whereas the IOM has a whole function as a longitudinal stabilizer, preventing the proximal migration of the radius. Kitamura et al. (2011) [9] emphasized that, although the TFCC is the primary soft-tissue stabilizer, specimens with a DOB showed significantly greater DRUJ stability in the neutral position than those without. They further reported that DRUJ laxity depends on the presence and thickness of the DOB, indicating that the existence of a DOB substantially influences DRUJ stability. Clinically, these findings reinforce the relevance of the DOB in surgical planning, as DOB reconstruction is indicated in patients with chronic symptomatic DRUJ instability who have failed nonsurgical modalities such as activity modification, orthosis fabrication, and injection therapies [13].

Although previous studies have described the prevalence and general attachments of the DOB, the precise three-dimensional pattern of its distal insertions remains unclear. In particular, its potential integration with the articular disc and the extensor carpi ulnaris tendon sheath—components of the TFCC and stabilizers of the DRUJ—has not been clearly reported.

Accordingly, the present study aimed to clarify the morphology and distal attachments of interosseous structures proximal to the DRUJ, focusing on both the distal oblique bundle (DOB), when present, and, in its absence, the membranous thickening of the distal IOM. By characterizing their three-dimensional course and connections to palmar and dorsal stabilizing structures, this study intended to refine the anatomical understanding of the distal IOM and to provide clinically relevant insights into DRUJ stabilization and surgical reconstruction.

## 2. Materials and Methods

The IOM of the forearm and wrist was examined in 48 specimens from 24 embalmed Korean cadavers (14 males, 10 females; mean age, 78.5 years). Of these, 46 specimens from 23 cadavers were dissected to assess the presence or absence of the DOB, and to document its morphology, fibre orientation, and attachment sites. In specimens without a DOB, the distal IOM proximal to the DRUJ was examined to identify structural features extending distally in place of the DOB. After removal of the forearm muscles, the IOM, DRUJ capsule, and articular disc were exposed for analysis.

The attachment levels of distal structures were measured in 42 specimens from 21 cadavers. For a DOB that appeared as a thickened fibrous band, the level was defined as the distance from its distal attachment on the margo interosseus ulnae to the most inferior point of the ulnar head. In specimens without a DOB, the vertical level of the membranous thickening of the IOM was defined as the distance from the thickened site to the most inferior point of the ulnar head. Measurements were obtained using the Digital Electronic Caliper (Fine Science Tools, Heidelberg, Germany). All measurements were performed by a single observer to maintain consistency.

To investigate the relationship between these distal structures and the articular disc of the DRUJ, the palmar capsule of the DRUJ was excised in 38 specimens from 19 cadavers. The articular disc was then inspected to determine whether the distal structures extended toward and attached to it.

The Mann–Whitney U test was used to compare the two groups because the data did not meet normality and variance assumptions. Welch’s *t*-test was also applied for reference, with statistical significance set at *p* < 0.05.

In addition, serial sectioned images of the distal forearm from one cadaver were analyzed to further confirm the anatomical relationships of the DOB and surrounding tissues. These images were obtained from a 71-year-old male cadaver, published in 2021 as part of a whole-body dataset [22]. The original images had a resolution of 8688 × 5792 pixels, a pixel size of 0.06 × 0.06 mm, a slice interval of 0.5 mm, and a 48-bit colour depth. For the present study, only images from the distal forearm to the wrist of the left side were selected, and all other regions were cropped. The resulting images had a reduced resolution of 2687 × 3543 pixels, while the pixel size, slice interval, and colour depth remained unchanged.

All cadavers were legally donated to the Daegu Catholic University School of Medicine and Dongguk University School of Medicine. The study was conducted in accordance with the Declaration of Helsinki. Written informed consent for body donation and its use in education and research was obtained from all donors or their next of kin. No donor was from a vulnerable population. The study protocol was approved by the Institutional Review Board of Daegu Catholic University (IRB No. CR-24-053-L; approval date: 5 June 2024).

## 3. Results

**Figure 1:**
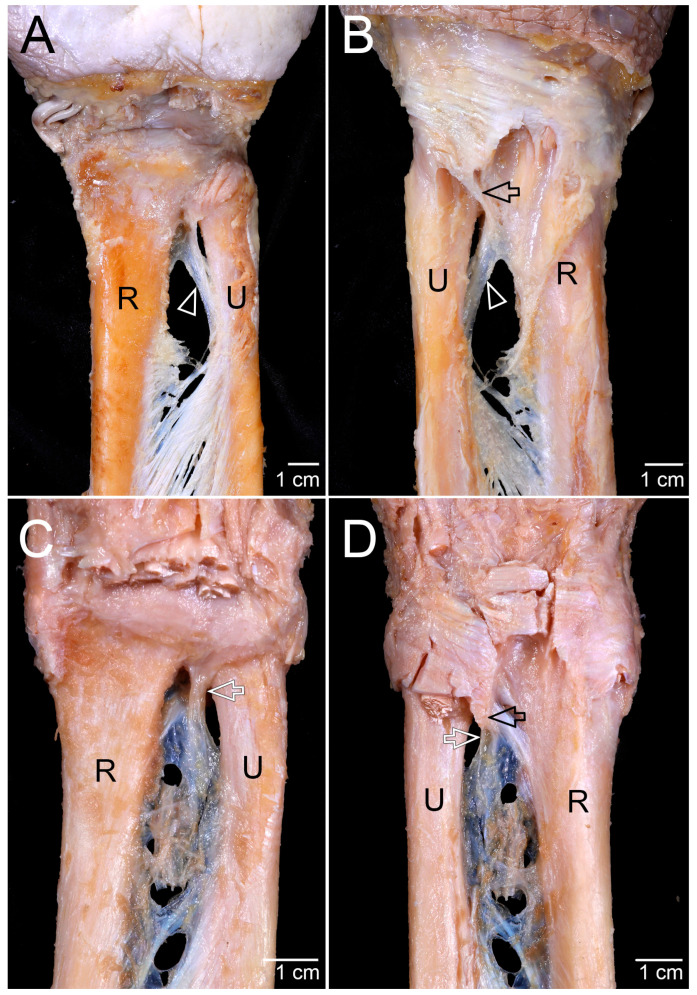
Morphological patterns of the distal interosseous structures proximal to the distal radioulnar joint (DRUJ): (**A**) When the distal oblique bundle (DOB, white arrowhead) was present, it originated from the interosseous border of the distal ulna and coursed obliquely toward the distal radius just proximal to the DRUJ. Before insertion, it gradually slanted both anteriorly and posteriorly and fanned out as it approached the DRUJ. The DOB inserted into several structures, including the distal radius, sigmoid notch of the radius, DRUJ capsule, and palmar radioulnar ligament on the palmar side. (**B**) On the dorsal side, the DOB (white arrowhead) extended toward the fibrous septum (black arrow) located between the fifth dorsal compartment (extensor digiti minimi tendon) and the sixth dorsal compartment (extensor carpi ulnaris tendon). (**C**) When the DOB was absent, a membranous thickening (white arrow) of the interosseous membrane was observed just proximal to the DRUJ. This thickening descended vertically with a fanning configuration, extending anteriorly to the DRUJ capsule and the palmar radioulnar ligaments. (**D**) Posteriorly, the membranous thickening (white arrow) extended toward the fibrous septum (black arrow) between the fifth and sixth dorsal compartments. R, radius; U, ulna.

In the distal IOM proximal to the DRUJ, two morphological patterns were observed: a thickened fibrous band corresponding to the DOB or, when absent, a membranous thickening of the IOM. The DOB was identified in 21 of 46 specimens (45.7%) from 23 cadavers (12 females, 9 males). When present, the DOB appeared either as a thickened band separated from the membranous portion of the distal IOM or as a thickened bundle within it. It originated from the interosseous border of the distal ulna and coursed obliquely toward the distal radius just proximal to the DRUJ. Before insertion, it gradually slanted both anteriorly and posteriorly and fanned out as it approached the joint. On the palmar side, the DOB inserted into the distal radius, the sigmoid notch of the radius, the DRUJ capsule, and the palmar radioulnar ligament, and in some specimens also into the periphery of the articular cartilage of the ulnar head. On the dorsal side, it extended to the fibrous septum between the fifth dorsal compartment (extensor digiti minimi tendon) and the sixth dorsal compartment (extensor carpi ulnaris tendon) (Figure 1A,B). Of the cadavers examined, 8 showed bilateral DOBs (34.8%), 5 showed unilateral DOBs (21.7%), and 10 had no DOB in either forearm (43.5%).

The mean attachment level of the DOB from the interosseous border of the ulna to the most inferior point of the ulnar head was 39.1 ± 9.7 mm (range, 23.7–58.1 mm), with mean of 39.9 ± 9.9 mm in females and 37.9 ± 10.1 mm in males. In cadavers with bilateral DOBs, the side-to-side difference in attachment level mean 10.6 mm (range, 1.3–22.4 mm).

In the remaining 25 specimens (54.3%, 8 females and 17 males), the DOB was absent. Instead, the distal IOM formed a membranous thickening that descended vertically just proximal to the DRUJ. This membranous thickening exhibited a fanning configuration, extending anteriorly to the DRUJ capsule and the palmar radioulnar ligaments, and dorsally to the fibrous septum between the fifth and sixth dorsal compartments, corresponding to the tendon sheath of the extensor carpi ulnaris (Figure 1C,D). The mean vertical level of this structure was 25.4 ± 4.8 mm (range, 19.1–39.6 mm), with mean values 27.4 ± 4.6 mm in females and of 24.5 ± 4.8 mm in males.

The DOB group (39.1 ± 9.7 mm, *n* = 18) showed significantly higher values than the membranous thickening of the IOM group (25.4 ± 4.8 mm, *n* = 24) (Mann–Whitney U = 39.0, *p* < 0.001; Welch’s *t* = −5.52, *p* < 0.001).

**Figure 2:**
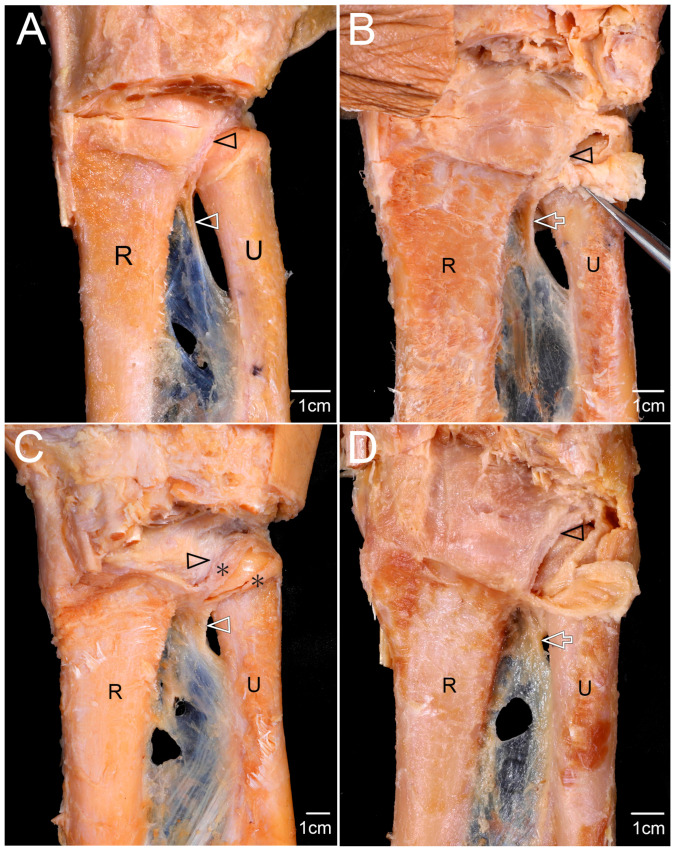
Attachment of distal interosseous structures to the articular disc of the distal radioulnar joint (DRUJ). (**A**) The DOB (white arrowhead) was attached to the proximal anterior margin of the articular disc (black arrowhead). The palmar radioulnar capsule was removed. (**B**) In specimens lacking a DOB, a membranous thickening (white arrow) of the distal interosseous membrane was attached to the proximal anterior portion of the articular disc (black arrowhead). The palmar radioulnar capsule was partially incised and reflected to expose the disc. (**C**) The DOB (white arrowhead) was not attached to the disc (black arrowhead), but extended toward the periphery of the articular cartilage of the ulnar head (asterisks). The palmar radioulnar capsule was partially removed to expose the cartilage. (**D**) The membranous thickening (white arrow) of the distal interosseous membrane was attached to the palmar radioulnar joint capsule, without connection to the articular disc (black arrowhead). The capsule was partially incised and reflected. R, radius; U, ulna.

In 26 of 38 specimens (68.4%), the distal structure—either a DOB or a membranous thickening—extended and attached to the proximal palmar portion of the articular disc of the DRUJ (Figure 2). Specifically, the DOB attached to the disc in 10 specimens (26.3%), whereas a membranous thickening was attached in 16 specimens (42.1%). The extent of attachment varied with the degree to which the structure projected toward the disc which was interposed between the distal radius and ulnar head (Table 1).

**Figure 3:**
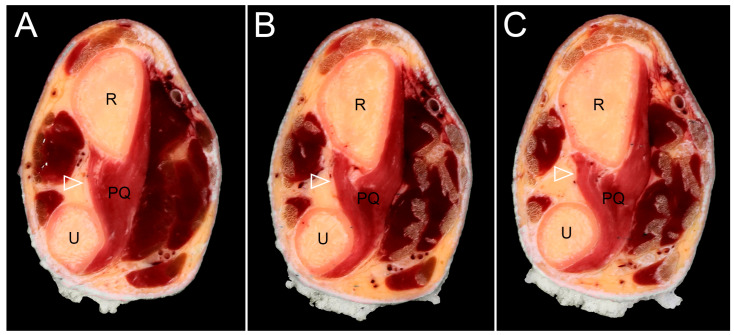
Serial transverse sections of the distal forearm. From proximal to distal (**A**–**C**), the distal interosseous membrane (arrowheads) gradually assumed an oblique orientation as it approached the distal radioulnar joint (DRUJ). This slanting plane corresponded to the distal structural change in the membrane, which formed either the DOB or a membranous thickening. PQ, pronator quadratus; R, radius; U, ulna.

Serial transverse sections confirmed the distal specialization of the IOM. From proximal to distal, the plane of the membrane became progressively oblique as it approached the DRUJ (Figure 3). This oblique orientation corresponded to the region where either a DOB or a membranous thickening was present, reflecting the fanning configuration observed in dissection. The membrane slanted anteriorly toward the palmar capsule and articular disc and posteriorly toward the dorsal septum.

## 4. Discussion

The DOB was identified in 45.7% of specimens in the present study, which is included within the 29–87.5% ranged reported in previous studies [5,8,9,12,14,15,16,17,18,19,20,21]. The remaining 54.3% of specimens demonstrated a membranous thickening proximal to the DRUJ, which likely represents cases without a DOB described in previous studies.

Despite morphological differences, both the DOB and the membranous thickenings showed similar attachment sites: anteriorly to the palmar joint capsule, articular disc, distal shaft of the radius, sigmoid notch of the radius, and posteriorly to the fibrous septum between the fifth (extensor digiti minimi tendon) and sixth (extensor carpi ulnaris tendon) dorsal compartments. The newly demonstrated fanning configuration of the DOB, slanting anteriorly toward the palmar capsule and posteriorly toward the dorsal septum and the extensor carpi ulnaris tendon sheath (a component of the TFCC), suggests that this structure consistently anchors to both palmar and dorsal components of the TFCC as well as to other stabilizers of the DRUJ, thereby engaging both volar and dorsal stabilizing elements of the joint.

Another notable finding of the present study was that the DOB or membranous thickening inserted into the articular disc in 68.4%, indicating direct integration with the TFCC. In such cases, the reciprocal tension of the DOB during forearm rotation could be transmitted directly to the disc, thereby reinforcing resistance to palmar and dorsal translation of the radius at the DRUJ. This attachment also provides an alternative pathway for load distribution, whereby axial forces transmitted through the radius can be transferred to the ulna via the disc in addition to the central band. Thus, the DOB may function not merely as a distal specialization of the IOM, but as an integral component of the TFCC contributing to both dynamic stabilization and load transfer in the distal forearm.

In the present study, both the DOB and the membranous thickening were also found to attach directly to the TFCC and other stabilizing structures of the DRUJ. The TFCC is composed of the articular disc, dorsal and palmar radioulnar ligaments, meniscus homologue, ulnar collateral ligament, and extensor carpi ulnaris tendon sheath [23], and it functions both as a cushion for the ulnar carpus and as a major stabilizer of the DRUJ. In addition, DRUJ stability is supported by intrinsic stabilizers—such as the radio-ulnar capsule and dorsal and palmar radioulnar ligaments—and extrinsic stabilizers, including the DOB, extensor carpi ulnaris tendon, pronator quadratus, and sheath of the sixth dorsal compartment which overlies extensor carpi ulnaris [6]. Taken together, these findings indicate that the DOB and the membranous thickening are not merely adjacent structures but integral components of this stabilizing system, directly reinforcing joint stability and contributing to the maintenance of congruency during forearm rotation.

Previous studies have reported variable measurements of the DOB, mainly due to differences in reference points and evaluation methods. In this study, the attachment level of the DOB—from the margo interosseus ulnae to the most inferior point of the ulnar head—was mean 39.1 mm, which lay within the range reported in the literature. According to Noda et al. (2009) [5], the DOB occupied 15% of the ulnar length with a mean width of 4.4 mm and thickness of 1.5 mm, whereas Angelis et al. (2023) [20] reported mean dimensions of 25.7 mm in length, 5.2 mm in width, and 0.9 mm in thickness. Kitamura et al. (2011) [9] found that the DOB originated from the distal ulna at a mean of 54 mm proximal to the ulnar head and that the distal IOM was significantly thicker in specimens with a DOB (mean 1.2 mm) than in those without (mean 0.4 mm). Hohenberger et al. (2018) [15] reported a mean DOB length of 24 mm, with ulnar and radial attachments located 35–48 mm and 25–35 mm, respectively, from the styloid processes, and a mean thickness of 0.9 mm. (Table 2).

Okada et al. (2014) [12] reported that 4 of 5 patients with chronic DRUJ instability showed absence of a DOB. Similarly, Moritomo (2012) [10] suggested that a thick DOB plays an important role in stabilizing the ulnar stump after the Sauvé-Kapandji procedure, and Kitamura et al. (2011) [9] demonstrated a significant correlation between DRUJ stability and DIOM thickness in the neutral position. Moritomo et al. (2009) [8] further described the DOB as an isometric stabilizer of the forearm.

Consistent with these clinical observations, our anatomical findings indicate that, when present, the DOB functions as a dynamic stabilizer, resisting palmar translation of the radius in pronation and dorsal translation in supination. In contrast, when the DOB was absent, a thickened membranous distal IOM appeared to provide a compensatory but less effective stabilizing role. This morphological variability highlights the complementary contributions of distal interosseous structures in constraining DRUJ laxity during forearm rotation.

The clinical significance of this variability is further reinforced by Martínez-Martínez et al. (2020) [24], who reported successful DOB reconstruction using an extensor carpi radialis longus hemitendon in patients with irreparable TFCC lesions, thereby restoring DRUJ stability and alleviating pain. These results support the concept that the DOB serves not only as an anatomical reinforcement of the distal IOM but also as a potential surgical target when primary stabilizers are deficient.

The transverse sections in the present study demonstrated that the distal IOM had an oblique orientation, corresponding to the trajectory of the DOB or membranous thickening. Rather than a simple continuation of the shaft-level membrane, this oblique configuration directed fibres palmarly to the palmar capsule and articular disc and dorsally to the septum between the extensor compartments. Such an arrangement links the volar and dorsal components of the TFCC, contributing to joint congruency and facilitating load transfer during forearm rotation.

From a biomechanical perspective, the two structural patterns of the distal IOM—either a DOB or a membranous thickening—likely account for individual differences in DRUJ stability and may contribute to variable surgical outcomes when this structure is overlooked. Surgeons should therefore be aware of these morphological patterns, preserving them when possible or reconstructing them when deficient. Conceptually, the close integration of the distal IOM with the TFCC supports its consideration not as an isolated ligament but as an extension of the TFCC.

The DOB and membranous thickening of the distal IOM may have important surgical implications. A well-developed DOB may act as a stabilizer against dorsal translation during pronation, whereas a membranous thickening may provide additional reinforcement when the DOB is absent or attenuated. Understanding these configurations is essential for accurate imaging interpretation and surgical planning. Biomechanical studies further support the stabilizing role of the DOB. Riggenbach et al. (2013) [25] reported that DOB reconstruction not only restored but even exceeded native stability and achieved comparable results to distal radioulnar ligament reconstruction, confirming its role as a dorsal stabilizer during pronation. Jawahier et al. (2023) [26] demonstrated that DOB augmentation using a TightRope system resulted in favourable outcomes with low recurrence rates, while de Vries et al. (2017) [27] showed that percutaneous suture-button fixation along the DOB axis effectively reduced dorsal translation in cadaveric DRUJ instability. Conversely, Hohenberger et al. (2023) [28] found no significant biomechanical benefit of DOB reconstruction when the TFCC was intact, suggesting that surgical restoration of the DOB may be most relevant when both the TFCC and distal IOM are compromised. Collectively, understanding the prevalence, attachment level, and structural variation in the DOB and membranous thickening provides not only morphological insight but also a practical anatomical foundation for both open and minimally invasive approaches to DRUJ stabilization.

This study has some limitations. First, although the present findings distinguish clearly between the fibrous band-like distal oblique bundle and the membranous thickening of the distal IOM, no histological confirmation was performed. Further histological validation will be necessary to confirm whether these structural differences correspond to distinct connective tissue organizations or represent variations within a continuous fibrous spectrum. Second, all specimens were derived from Korean cadavers, which may limit the generalizability of the results to other populations. Anatomical variations in the distal IOM and its distal specializations may differ among ethnic groups. Future comparative studies involving multiethnic samples, combined with histological and imaging analyses, would help to reinforce and expand the anatomical interpretations presented in this study.

Ultimately, a detailed understanding of the distal IOM—its morphological patterns, attachment sites, and biomechanical functions—offers not only an expanded anatomical framework but also practical guidance for surgical management, with the potential to improve outcomes and reduce chronic DRUJ instability after trauma or reconstruction.

## Figures and Tables

**Table 1:** Morphological patterns of the distal interosseous structures proximal to the distal radioulnar joint (DRUJ).

Parameter	Distal Oblique Bundle	Membranous Thickening of Distal Interosseous Membrane (IOM)
Prevalence (*n* = 46 specimens from 23 cadavers)	21 specimens (45.7%)	25 specimens (54.3%)
Sex distribution (males 13 cadavers, females 10 cadavers)	Male: 9 specimens	Male: 17 specimens
	Female: 12 specimens	Female: 8 specimens
Origin	Margo interosseus ulnae	Distal IOM proximal to DRUJ
Insertion	Distal radius, sigmoid notch of the radius, DRUJ capsule, palmar radioulnar ligament, occasionally to the articular disc; dorsally to fibrous septum between 5th and 6th dorsal compartments	DRUJ capsule, palmar radioulnar ligament, occasionally to the articular disc; dorsally to fibrous septum between 5th and 6th dorsal compartments
Mean attachment level (mm, overall; *n* = 42 specimens)	From the margo interosseus ulnae to the most inferior point of the ulnar head: 39.1 ± 9.7 (range, 23.7–58.1)	From the thickened site of the IOM to the most inferior point of the ulnar head: 25.4 ± 4.8 (range, 19.1–39.6)
Mean attachment level (mm, females)	39.9 ± 9.9	27.4 ± 4.6
Mean attachment level (mm, males)	37.9 ± 10.1	24.5 ± 4.8
Attachment to articular disc (*n* = 38 specimens)	10 specimens (26.3%)	16 specimens (42.1%)

**Table 2:** Comparative summary of previously reported findings on the distal oblique bundle (DOB).

Study	Type	Prevalence	Mean Length or Ulnar Attachment Level (to Ulnar Head or Styloid Process)	Mean Width (mm)	Mean Thickness (mm)
Noda et al. (2009) [5]	Embalmed cadavers	12 of 30 specimens (40%)	15% of ulnar length (from ulnar attachment to the ulnar head); 9.9% of radial length (from the radial attachment to the radial styloid process)	4.4	1.5
Kitamura et al. (2011) [9]	Fresh-frozen cadavers	4 of 10 specimens (40%)	54 mm (from ulnar attachment of the DOB to the ulnar styloid process); 35 mm (radial attachment of the DOB to the radial styloid process)	–	1.2 (0.4 without the DOB)
Dy et al. (2014) [21]	Fresh-frozen cadavers	5 of 10 specimens (50%)	–	–	0.9
Okada et al. (2014) [12]	Intraoperative	10 of 15 cases (67%)	–	–	1.1 (0.3 without DOB)
Kim et al. (2017) [14]	MRI	26 of 80 cases (32.5%)	–	–	1.4 (0.6 without DOB)
Hohenberger et al. (2018) [15]	Embalmed cadavers	53 of 200 specimens (29%)	48 mm (proximal ulnar attachment); 35 mm (distal ulnar attachment) from ulnar styloid process; 24 mm (ulna–radius interval, mid-bundle level)	9	0.9
Angelis et al. (2023) [20]	Fresh-frozen cadavers	11 of 28 specimens (39.3%)	25.7 mm (from midpoint of ulnar attachment to midpoint of radial insertion)	5.2	0.9
Present study (2025)	Embalmed cadavers	21 of 46 specimens (45.7%)	39.1 ± 9.7 mm (from the distal ulnar attachment to ulnar head)	–	–

## Data Availability

The data presented in this study is available on request from the corresponding author.
